# Dapagliflozin in Patients with Chronic Heart Failure: A Systematic Review and Meta-Analysis

**DOI:** 10.1155/2021/6657380

**Published:** 2021-03-30

**Authors:** Ru-ping Cai, Yu-li Xu, Qiang Su

**Affiliations:** Department of Cardiology, Affiliated Hospital of Guilin Medical University, Guilin, China

## Abstract

Sodium-glucose cotransporter-2 (SGLT2) inhibitors represent newly developed oral antidiabetic drugs that are practiced for type 2 diabetes mellitus management and may decrease the risk of the first hospitalization in heart failure. The activity of SGLT2 inhibitors is not related to glucose, and the effectiveness and safety of SGLT2 inhibitors in individuals with chronic heart failure (CHF) remain unclear. We systematically retrieved PubMed, Cochrane Library, Embase, NCKI, VIP, Wanfang Data, and ClinicalTrials.gov records to identify eligible trials. The primary endpoints were cardiovascular death/hospitalization for heart failure (CV death/HHF), cardiovascular death, and hospitalization for heart failure. Secondary endpoints included hypoglycemia, volume depletion, urinary tract infection, left ventricular ejection fraction (LVEF), and NT-proBNP. Nine randomized controlled clinical trials were included. Dapagliflozin was reported to significantly decrease CV death/HHF (relative risk (RR): 0.75; 95% confidence interval (CI): 0.68–0.84), CV death (RR: 0.80; 95% CI: 0.68–0.93), and HHF (RR: 0.72; 95% CI: 0.63–0.83). There was no effect on hypoglycemia (RR: 0.69; 95% CI: 0.34–1.40), volume depletion (RR: 1.17; 95% CI: 0.97–1.41), urinary tract infection (RR: 0.82; 95% CI: 0.43–1.57), LVEF (WMD: 0.53; 95% CI: −4.04–5.09), or NT-proBNP (SMD: −0.66; 95% CI: −1.42–0.10). The risk of CV death/HHF, CV death, and HHF was lower among patients receiving dapagliflozin than patients receiving placebo.

## 1. Introduction

According to ESC guidelines, HF is a clinical syndrome characterized by typical symptoms (e.g., breathlessness, ankle swelling, and fatigue) that may be accompanied by signs (e.g., elevated jugular venous pressure, pulmonary crackles, and peripheral oedema) caused by a structural and/or functional cardiac abnormality, resulting in a reduced cardiac output and/or elevated intracardiac pressures at rest or during stress [1]. Despite advances in medicine, chronic heart failure, as a fatal disease, has always been a challenge for healthcare providers. Globally, around 64 million people suffer from CHF (half of whom have reduced ejection fraction). CHF is a chronic progressive disease, and 50% of patients die within five years after diagnosis [2]. CHF is the major cause of hospitalization for people over 65 years of age and is correlated with significant healthcare and economic burden on nations globally. Due to the use of aldosterone receptor antagonists, angiotensin-converting enzyme inhibitors (ACEIs), *β*-blockers, and angiotensin receptor blockers (ARBs), CHF evolved from a fatal disease to a severe disease that needs long-term team management [3]. Results of the recently conducted clinical studies have shown that sodium-glucose cotransporter-2 (SGLT2) inhibitors can reduce the risk of CHF in individuals having type 2 diabetes mellitus (T2DM) [4–9]. Recent clinical trials have shown that the SGLT2 inhibitor dapagliflozin can reduce the cardiovascular death/hospitalization for heart failure (CV death/HHF) in patients with CHF, whether or not they have T2DM [10–12]. Recently, the FDA authorized dapagliflozin for adult patients with HFrEF (with or without T2DM) to reduce the risk of cardiovascular deaths and hospitalization due to HF and associated disorders. This suggests that dapagliflozin may become a cornerstone and a candidate for the treatment of HF, in addition to angiotensin-converting enzyme inhibitors (ACEIs), angiotensin receptor/neprilysin inhibitors (ARNIs), beta-blockers, and mineralocorticoid receptor antagonists (MRAs). Although some clinical trials have demonstrated the effect of dapagliflozin in avoiding HF, its efficacy and clinical application remain controversial and debatable, and there is no standardized clinical guideline for reference. Accordingly, we used the Cochrane systematic review methodology to objectively appraise studies that analyzed dapagliflozin in preventing and treating CHF, intending to provide a scientific basis for clinical use.

## 2. Methods

### 2.1. Search Strategy

We systematically retrieved records from PubMed, Cochrane Library, Embase, NCKI, VIP, Wanfang Data, and ClinicalTrials.gov that were published before November 30, 2020, which contained one or more of the following keywords: dapagliflozin; Forxiga; Chronic Heart Failure; Myocardial Failure; Right-Sided; Systolic Heart Failure.; Sodium-Glucose Transporter 2 Inhibitors; (2S, 3R, 4R, 5S, 6R)-2-(4-chloro-3-(4-ethoxybenzyl)phenyl)-6-(hydroxymethyl)tetrahydro-2H-pyran-3,4,5-triol/BMS-512148; Heart Decompensation; Left-Sided; Heart Failure.

No language restrictions were imposed. Besides, references from retained articles were manually searched to find other potentially pertinent data. The protocol was registered with PROSPERO (CRD42020215216).

### 2.2. Study Inclusion and Exclusion Criteria

  Inclusion criteria:
  All studies are randomized controlled trials (RCTs). The included patients conform to the following criteria: evidence of structural heart disease and manifestation of circulating congestion, age ≥18 years old, ejection fraction ≤50%, New York Heart Association (NYHA) class ≥ I, and NT-proBNP ≥ 600 pg per milliliter (or ≥400 pg per milliliter if they had been hospitalized for heart failure within the previous 12 months).
  Exclusion criteria:
  Observational studies, diagnostic studies, and animal experiments were excluded, type 1 diabetes mellitus, estimated glomerular filtration rate (eGFR) <30 ml/min/1.73 m^2^, systolic blood pressure <95 mm Hg, acute or previous myocardial infarction, moderate-to-severe liver, and kidney dysfunction.
  All articles were independently reviewed using predefined inclusion and exclusion criteria. Initial inclusion/exclusion of articles was based on titles and abstracts, followed by full-text review in cases of uncertainty. Studies with a follow-up time of <12 weeks were excluded, as well as those that did not report any events of interest. In cases where several publications were based on a single study, we selected the RCT that included the longest follow-up time. Any discrepancies regarding inclusion or exclusion were settled by discussion. If any doubts remained, the third investigator (Qiang Su) made the final decision.

### 2.3. Data Extraction

Two researchers (Cai Ru-ping and Xu Yu-li) used standardized forms to collect data in duplicate. In cases of discrepancy, all authors discussed the results and established a consensus. The primary endpoints were acquired from the original published manuscript: cardiovascular death/hospitalization for heart failure (CV death/HHF), cardiovascular death, and hospitalization for heart failure; secondary endpoints: hypoglycemia, volume depletion, urinary tract infection, left ventricular ejection fraction (LVEF), and NT-proBNP. For each study, we searched the registration number, the type of the study, the data source of the primary endpoint, the drugs received by the control group, the follow-up time, and the dose (mg) of dapagliflozin. For the experimental group and control group in each study, we collected baseline characteristics (Table 1).

### 2.4. Quality Assessment

The consistency of the RCTs was measured using the prejudice risk evaluation method described in Cochrane Handbook 5.1.0 [19], and the following criteria have been evaluated: random sequence generation, allocation concealment, personnel, patient blindness, incomplete outcome data, selective reporting, the blindness of outcomes' assessment, and other biases. Studies were determined to have a strong degree of high, low, or unclear, based on the above 7 objects.

### 2.5. Statistical Analysis

Meta-analysis was implemented using RevMan 5.3 software. Relative risk (RR) was used for the analysis of efficacy, while each effect size was denoted as a 95% confidence interval (CI). A *p* value was used for finding out heterogeneity between studies, and *p* < 0.1 represented a significant degree of heterogeneity. *I*^2^ > 75%, *I*^2^ > 50%, and *I*^2^ > 25% represented high, moderate, and low heterogeneity between studies, respectively. Where there was statistical homogeneity between the studies (*p* > 0.1, I^2^ < 50%), the fixed-effect model was used; else, the model of random effects was adopted. We will have subgroups about the effect of heart function on results.

## 3. Results

### 3.1. Search Results

**Figure 1:**
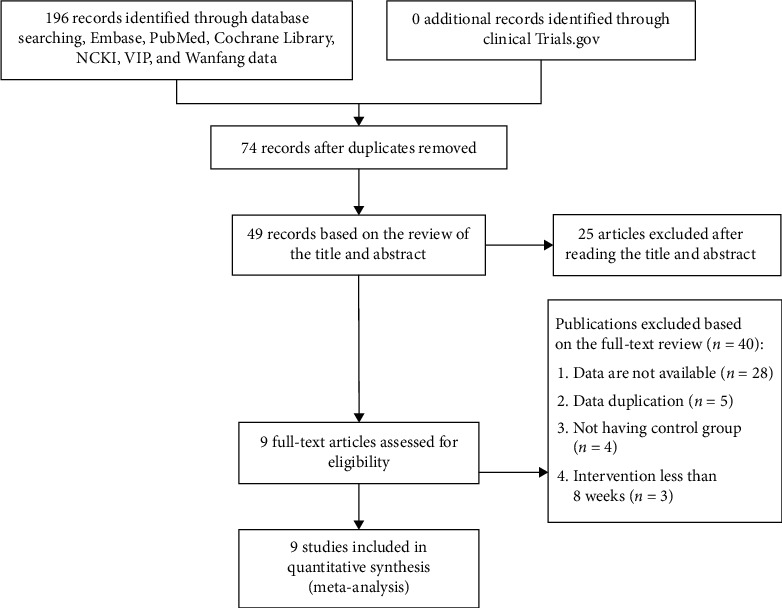
Flowchart of the study selection.

Through electronic retrieval, we identified and reviewed 1021 studies from the aforementioned sources as shown in Figure 1. The main reasons for their exclusion included the unavailable data (34 cases), duplicate data (8 cases), not having control group (7 cases), and short duration (3 cases). Overall, 9 studies [10–18] were included representing a total of 6278 patients (randomly divided into 3125 patients in the group of dapagliflozin and 3153 patients in the control group). 1108 patients with CV death/HHF, 592 cardiovascular deaths, 710 hospitalization for heart failure, 35 hypoglycemia, 402 cases of volume depletion, 38 patients with urinary tract infections, 602 LVEF, and 410 NT-proBNP were reported (in total).

### 3.2. Research Characteristics and Risk-of-Bias Assessment

**Figure 2:**
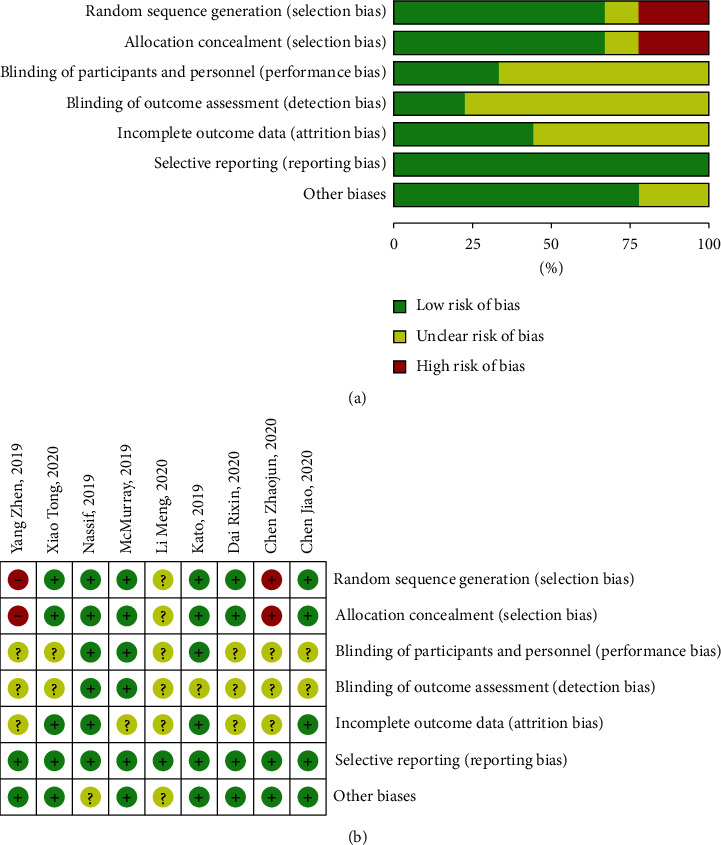
(a) “Risk of bias” graph: review authors' judgements about each risk of bias item presented as percentages across all included studies. (b) “Risk of bias” summary: review authors' judgements about each risk of bias item for each included study.

The trials which were included in the study were published before May 1, 2020. The duration varies from 12 weeks to 4 years. Baseline characteristics by CHF patients are summarized in Table 1, which also provides detailed information about the included studies. Six studies [10–13, 15, 17] used computer randomization, and 3 studies [14, 16, 18] did not use computer randomization resulting in high risks. The overall possibility of bias in the selected reports and other biases was low, and the incomplete outcome data were unclear in a few studies that were included. Detailed information on quality assessment is shown in Figure 2.

### 3.3. Meta-Analysis

#### 3.3.1. The Effect of Dapagliflozin on Cardiovascular Death/Hospitalization for Heart Failure

**Figure 3:**
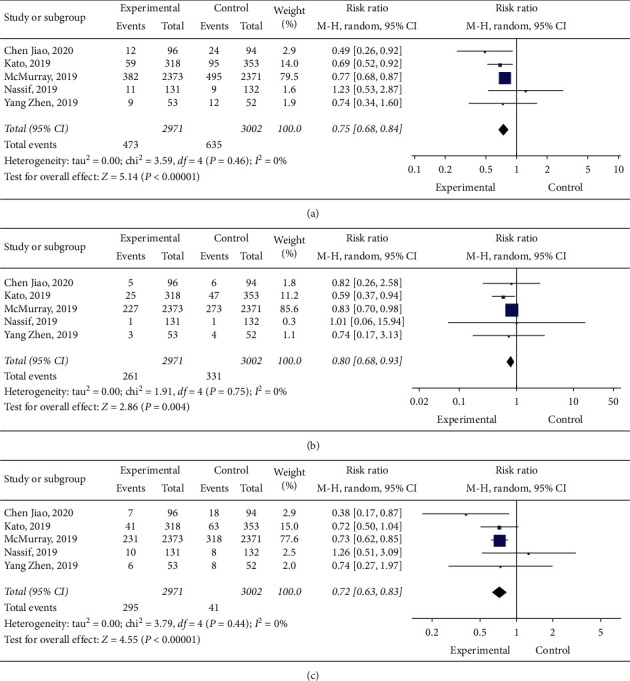
(a) Forest plot of the effect of dapagliflozin on cardiovascular death/hospitalization. (b) Forest plot of the effect of dapagliflozin on heart failure hospitalization. (c) Forest plot of the effect of dapagliflozin on cardiovascular death.

Five studies [10–13, 18] reported CV death/HHF, of which 473 patients received dapagliflozin and 635 patients received a placebo. No statistical heterogeneity among the studies existed (*I*^2^ = 0%; *p*=0.46). Using a model of random effects, we demonstrated that dapagliflozin highly decreased the prevalence of CV death/HHF (RR: 0.75, 95% CI: 0.68–0.84, and *p* < 0.00001) (Figure 3(a)).

#### 3.3.2. The Effect of Dapagliflozin on Cardiovascular Death

Five studies [10–13, 18] reported cardiovascular deaths, of which 261 patients received dapagliflozin and 331 patients received a placebo. There was no statistical heterogeneity between the studies (*I*^2^ = 0%; *p*=0.75), and using a random-effect model, we demonstrated that dapagliflozin significantly reduced cardiovascular deaths (RR: 0.80, 95% CI: 0.68–0.93, and *p*=0.004) (Figure 3(b)).

#### 3.3.3. The Effect of Dapagliflozin on Hospitalization for Heart Failure

Five studies [10–13, 18] reported hospitalization for heart failure, of which 295 patients received dapagliflozin and 415 patients received a placebo. There was no statistical heterogeneity between the studies (*I*^2^ = 0%; *p*=0.44), and using a random-effect model, we demonstrated that dapagliflozin significantly reduced hospitalization for heart failure (RR: 0.72, 95% CI: 0.63–0.83), and *p* < 0.00001) (Figure 3(c)).

#### 3.3.4. The Effect of Dapagliflozin on Hypoglycemia, Volume Depletion, Urinary Tract Infections, Left Ventricular Ejection Fraction, and NT-proBNP

**Figure 4:**
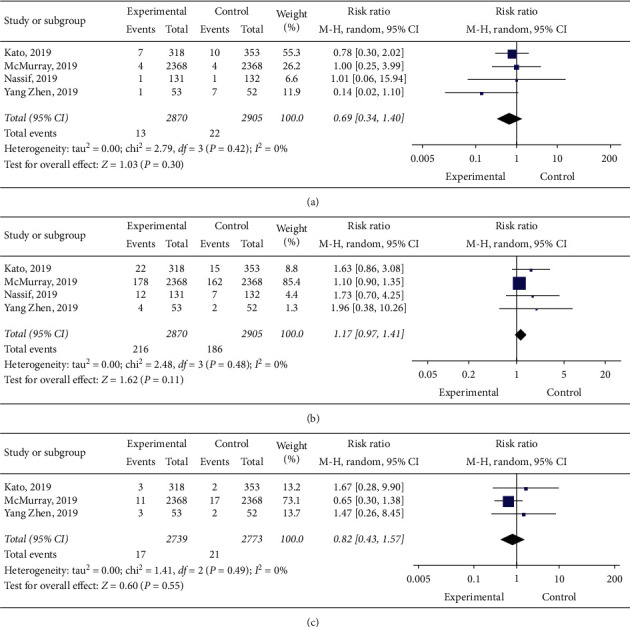
(a) Forest plot of the effect of dapagliflozin on hypoglycemia. (b) Forest plot of the effect of dapagliflozin on volume depletion. (c) Forest plot of the effect of dapagliflozin on urinary tract infection.

**Figure 5:**
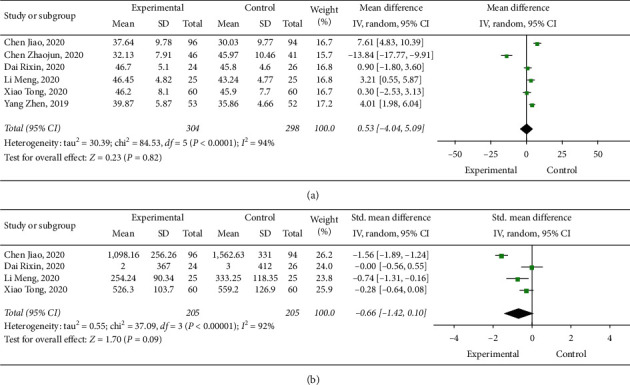
(a) Forest plot of the effect of dapagliflozin on left ventricular ejection fraction. (b) Forest plot of the effect of dapagliflozin on NT-proBNP.

Overall, 35 cases of hypoglycemia were stated across the studies, as well as 402 cases of volume depletion and 38 cases of urinary tract infections. Meta-analysis using a random-effect model revealed that dapagliflozin did not decrease the occurrence of hypoglycemia (RR: 0.69, 95% CI: 0.34–1.40, and *p*=0.3) (Figure 4(a)), volume depletion (RR: 1.17, 95% CI: 0.97–1.41, and *p*=0.11) (Figure 4(b)), and urinary tract infection (RR: 0.82, 95% CI: 0.43–1.57, and *p*=0.55) (Figure 4(c)). LVEF (WMD: 0.53, 95% CI: −4.04–5.09, and *p*=0.82) (Figure 5(a)) and NT-proBNP (SMD: −0.66, 95% CI: −1.42–0.10, and *p*=0.09) (Figure 5(b)) were not affected by dapagliflozin.

### 3.4. Subgroup Analysis about the Effect of Heart Function on Results

**Figure 6:**
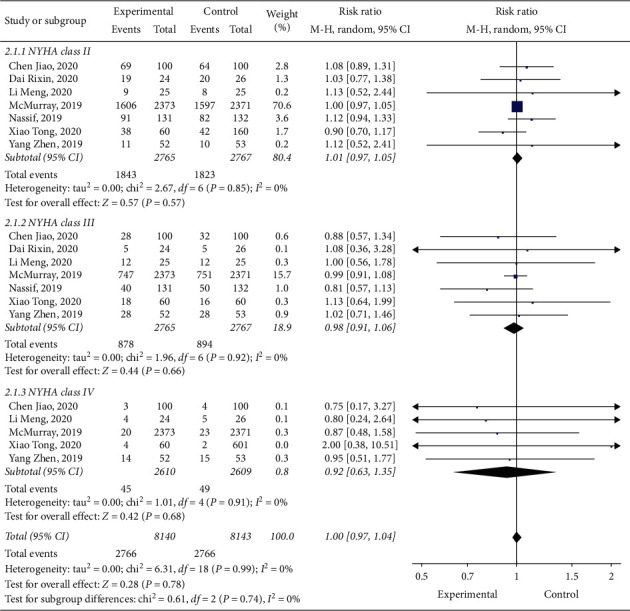
Subgroup analysis about the effect of heart function on results.

We performed a subgroup analysis based on the NYHA class, and 7 studies [10, 11, 13, 15–18] reported relevant data. No statistical heterogeneity was reported between studies (*I*^2^ = 0; *p*=0.74), and when a model of random effects was employed for analysis, we found that difference among the three groups was not statistically significant (RR: 1.00, 95% CI: 0.97–1.04, and *p*=0.78) (Figure 6). The positive effect of dapagliflozin on patients with HF may not be related to the degree of HF; therefore, dapagliflozin is suitable for patients with mild or severe HF.

### 3.5. Subgroup Analysis about HFrEF and HFmrEF

**Figure 7:**
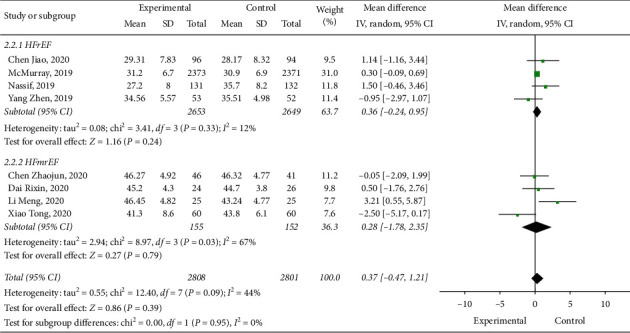
Subgroup analysis about HFrEF and HFmrEF.

We performed a subgroup analysis based on HFrEF and HFmrEF, and 8 studies [10, 11, 13–18] reported relevant data. No statistical heterogeneity was reported between studies (*I*^2^ = 0; *p*=0.39), and when a model of random effects was employed for analysis, we found that difference among the three groups was not statistically significant (WMD: 0.37, 95% CI: −0.47–1.21, and *p*=0.39) (Figure 7). The positive effect of dapagliflozin on patients with HF may not be related to the left ventricular ejection fraction; therefore, dapagliflozin is suitable for patients with HFrEF and HFmrEF.

### 3.6. Sensitivity Analyses and Reporting Bias

**Figure 8:**
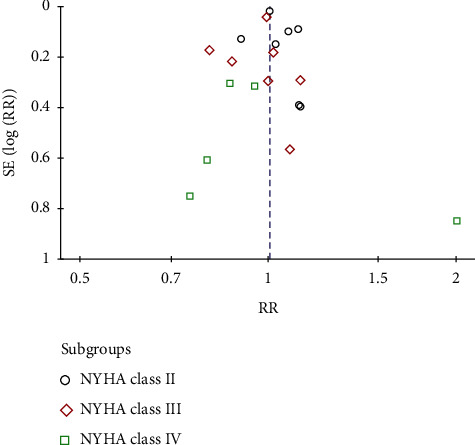
Funnel plot of subgroup analysis about the effect of heart function on results.

After excluding 2 studies [13, 15], we found that dapagliflozin can significantly improve NT-proBNP (RR: −0.45, 95% CI: −0.89, −0.02, and *p*=0.04) and heterogeneity (*p* > 0.1). It shows that these two studies [13, 15] are sources of heterogeneity. The funnel chart was almost symmetrical, and the reporting bias of the studies was relatively small (Figure 8).

## 4. Discussion

SGLT2 inhibitors, which act independently of insulin, selectively inhibit SGLT2 in the kidney and block the reabsorption of glucose, thereby lowering blood glucose levels. This process does not depend on insulin resistance and *β*-cell function [20]. Recent studies have shown that in addition to lowering blood glucose, the SGLT2 inhibitor dapagliflozin also has a positive effect on patients with heart failure [10–12, 21–23]. The FDA has recently approved dapagliflozin for adult patients with HFrEF (with or without T2DM) to decrease the danger of cardiovascular death and hospitalization for heart failure. However, its efficacy and clinical application are still controversial, and there are no standardized clinical guidelines for reference. Consequently, we thoroughly evaluated the literature to objectively appraise dapagliflozin.

The efficacy of dapagliflozin on the heart may be based on its ability to decrease preload of the heart by natriuresis and diuresis [24] and to decrease postload by reducing arterial blood pressure and changing vascular function [25]. SGLT2 inhibitors may also ameliorate and/or optimize cardiac energy metabolism and improve cardiac efficacy and cardiac output by increasing myocardial energy and substrate efficiency. Studies have shown that SGLT2 inhibitors may/could increase the production of ketone bodies and *β*-hydroxybutyrate. Cardiac uptake of carbohydrates (pyruvate, lactate, and glucose) was shown to be reduced, while uptake of the total *β*-hydroxybutyrate and ketone bodies was enhanced in diabetic patients when they were compared with nondiabetic patients. Consequently, ketone bodies may be used as a source of energy moderately substituting glucose in patients with diabetes [26–28]. This theory is supported by animal experiments, indicating that SGLT2 inhibitors may/could increase myocardial ketone body consumption and reduce cardiac glucose utilization and lactate production [29]. However, an in-depth analysis is required to confirm this possibility. At the same time, studies have found that SGLT2 inhibition can promote the degradation of branched-chain amino acids, thereby providing an energy source for myocardium in patients with T2DM and HF [30]. SGLT2 inhibitors are anti-inflammatory and have an inhibitory effect on cardiac fibrosis. Myocardial fibrosis is widely considered to be an important factor in the development of HF. Recently, some experimental data indicate that SGLT2 inhibits collagen fiber synthesis by augmenting the activation of M2 macrophages and restraining myofibroblast differentiation, thus showing significant antifibrosis effect [31].

In this meta-analysis, we quantitatively analyzed the efficacy and safety of dapagliflozin for the management of chronic heart failure. We showed that dapagliflozin can significantly decrease the frequency of cardiovascular death/hospitalization for heart failure, cardiovascular death, and hospitalization for heart failure. Furthermore, it improves survival and reduces the need for hospitalization. This suggests that dapagliflozin may provide an extra treatment option for patients with chronic heart failure in addition to ACEIs, ARNIs, beta-blockers, and MRAs. In summary, patients with HF should be treated with a combination of the following four drugs as early as possible: an ARNI, beta-blocker, MRA, and SGLT2 inhibitor. Volume depletion, hypoglycemia, and urinary tract infections are not affected by dapagliflozin. In the past, it was believed that patients with poorer heart function and left ventricular ejection fraction had a worse prognosis. However, subgroup analysis showed surprising results: the effect of dapagliflozin was not affected by heart function grades and LVEF. The positive effect of dapagliflozin in heart failure has nothing to do with the degree of heart function and LVEF. Therefore, dapagliflozin treatment is applicable in patients with mild and severe HF. Besides, dapagliflozin decreased the risk of death and deteriorating the HF and relieved symptoms among different ages of people [21]. Some researchers believe that the combination of new targeted drugs such as aldosterone receptor antagonists, angiotensin receptor-neprilysin inhibitors, and SGLT2 inhibitors could significantly decrease the risk of death in patients with heart failure. Some studies have also shown that dapagliflozin has a positive effect on blood pressure reduction, weight loss, left ventricular remodeling, reducing acute kidney injury, atrial fibrillation, and atrial flutter [15, 16]. The findings of our meta-analysis suggest that dapagliflozin has useful effects other than glucose lowering [9, 32–37]; thus, they potentially expand the therapeutic role of dapagliflozin beyond patients with diabetes.

Although dapagliflozin treatment carries several advantages, it also leads to some adverse reactions in clinical practice. Diabetic ketoacidosis (DKA) is a common complication in patients with diabetes, which is more common in patients with T1D than T2D patients. Several SGLT2 inhibitors have been FDA approved for the management of T1DM in the EU and Japan, but they have not yet been approved in the United States and China. In March and July 2019, respectively, sotagliflozin and dapagliflozin were rejected by the FDA for the treatment of T1DM, and in March 2020, empagliflozin was rejected because a higher number of patients in the experimental group developed DKA compared with that of the placebo group [38, 39]. Besides, some studies showed that dapagliflozin increased the incidence of rare and chronic infectivity of the genitals and surrounding areas, called as Fournier's gangrene (FG), which is a life-threatening fulminant infection that affects the subcutaneous tissue around the genitals. The disease is characterized by rapid onset and hyperthermia, extensive necrosis, and gangrene in the infected tissue [40, 41].

## 5. Limitations

This study has some limitations. The population that met the inclusion criteria was HFrEF and HFmrEF patients, and patients with preserved ejection fraction heart failure (HFpEF) may not be eligible for this drug. In this study, males accounted for 77% of the population, and black patients accounted for only 5.7%, which might affect the generalizability of the study data. However, to obtain further robust evidence to guide clinical practice, more extensive, multicenter, and high-quality clinical research must be performed.

## 6. Conclusion

We assessed the incidence of cardiovascular death/hospitalization for heart failure, cardiovascular death, and hospitalization for heart failure in patients with heart failure. Dapagliflozin significantly decreased the incidence of CV death/HHF (relative risk (RR): 0.75; 95% confidence interval (CI): 0.68–0.84), CV death (RR: 0.80; 95% CI: 0.68–0.93), and HHF (RR: 0.72; 95% CI: 0.63–0.83).

## Figures and Tables

**Table 1:** Randomized controlled trial studies included in the systematic review and meta-analysis.

Author	Year	Country	Design patients	Inclusion	Duration	Experimental			Control			Outcomes
						Intervention	*N*	Age	Intervention	*N*	Age	
Chen et al. [13]	2020	China	RCT	HFrEF + T2DM	24 w	DAPA 10 mg/d + RT	96	65.14 ± 9.24	Placebo + RT	94	66.37 ± 10.04	①②③⑦⑧
Chen et al. [14]	2020	China	RCT	HFmrEF + T2DM	12 w	DAPA 10 mg/d + RT	46	64.1 ± 6.3	Placebo + RT	41	63.7 ± 6.7	⑦
Dai et al. [15]	2020	China	RCT	HFmrEF + T2DM	24 W	DAPA 5 mg/d + RT	24	67 ± 6.8	Placebo + RT	26	66 ± 7.1	⑦⑧
Kato et al. [12]	2019	America	RCT	HFrEF + T2DM	219 w	DAPA 10 mg/d + RT	318	63	Placebo + RT	351	63	①②③④⑤⑥
Li [16]	2020	China	RCT	HFmrEF + T2DM	12 w	DAPA 5 mg/d + RT	25	58.37 ± 5.39	Placebo + RT	25	58.46 ± 5.41	⑦⑧
McMurray et al. [10]	2019	England	RCT	HFrEF	78 w	DAPA 10 mg/d + RT	2373	66.2 ± 11.0	Placebo + RT	2371	66.5 ± 10.8	①②③④⑤⑥
Nassif et al. [11]	2019	America	RCT	HFrEF	12 w	DAPA 10 mg/d + RT	131	62.2 ± 11.1	Placebo + RT	132	60.4 ± 12.0	①②③④⑤
Tong et al. [17]	2020	China	RCT	HFmrEF + T2DM	24 w	DAPA 10 mg/d + RT	60	70.2 ± 9.6	Placebo + RT	60	68.5 ± 5.8	⑦⑧
Yang et al. [18]	2019	China	RCT	HFrEF + T2DM	24 w	DAPA 5 mg/d + RT	52	66.31 ± 8.53	Placebo + RT	53	65.18 ± 8.37	①②③④⑤⑥⑦

① Cardiovascular death/hospitalization for heart failure (CV death/HHF), ② cardiovascular death, ③ hospitalization for heart failure, ④ hypoglycemia, ⑤ volume depletion, ⑥ urinary tract infection, ⑦ left ventricular ejection fraction (LVEF), and ⑧ NT-proBNP. RCT: randomized controlled trial; T2DM: type 2 diabetes; HFrEF: heart failure with reduced ejection fraction; HFmrEF: midrange ejection fraction heart failure; DAPA: dapagliflozin; RT: basic recommended therapy.

## Data Availability

1. The datasets generated and/or analyzed during the current study are available in the PubMed, Cochrane Library, Embase, NCKI, VIP, Wanfang Data, and ClinicalTrials.gov repository (https://pubmed.ncbi.nlm.nih.gov/, https://www.cochranelibrary.com/, https://www.embase.com/, https://www.cnki.net/, http://www.cqvip.com/, http://www.wanfangdata.com.cn/index.html, and https://www.clinicaltrials.gov/). 2. The datasets generated and/or analyzed during the current study are available from the corresponding author upon reasonable request. 3. All the data generated or analyzed during this study are included within this published article (and its supplementary information files).

## Abbreviations

SGLT2: Sodium-glucose cotransporter-2
CHF: Chronic heart failure
NCKI: China National Knowledge Infrastructure
CV death: Cardiovascular death
HHF: Hospitalization for heart failure
LVEF: Left ventricular ejection fraction
NT-proBNP: N-terminal pro-B-type natriuretic peptide
RR: Relative risk
CI: Confidence interval
T2DM: Type 2 diabetes mellitus
FDA: Food and Drug Administration
HF: Heart failure
HFrEF: Heart failure and reduced ejection fraction
RCTs: Randomized controlled trials
NYHA: New York Heart Association
eGFR: Estimated glomerular filtration rate
DKA: Diabetic ketoacidosis
T1DM: Type 1 diabetes mellitus
FG: Fournier's gangrene
HFmrEF: Midrange ejection fraction heart failure
HFpEF: Heart failure and preserved ejection fraction
DAPA: Dapagliflozin
RT: Recommended therapy
SD: Standard deviation
ACEIs: Angiotensin-converting enzyme inhibitors
ARNIs: Angiotensin receptor/neprilysin inhibitors
MRAs: Mineralocorticoid receptor antagonists.

## Supplementary Materials

Supplementary Materials Supplementary: PRISMA 2009 Checklist.
